# Barriers and facilitators to disease-modifying antirheumatic drug use in patients with inflammatory rheumatic diseases: a qualitative theory-based study

**DOI:** 10.1186/s12891-016-1289-z

**Published:** 2016-10-21

**Authors:** Marieke Voshaar, Johanna Vriezekolk, Sandra van Dulmen, Bart van den Bemt, Mart van de Laar

**Affiliations:** 1 Department Psychology, Health and Technology, University of Twente, Enschede, The Netherlands; 2 Department of Rheumatology, Sint Maartenskliniek, Nijmegen, The Netherlands; 3 NIVEL (Netherlands Institute for Health Services Research), Utrecht, The Netherlands; 4 Department of Primary and Community Care, Radboud University Medical Centre, Nijmegen, The Netherlands; 5 Faculty of Health Sciences, University College of Southeast Norway, Drammen, Norway; 6 Department of Pharmacy, Sint Maartenskliniek, Nijmegen, The Netherlands; 7 Department of Pharmacy, Radboudumc, Nijmegen, The Netherlands; 8 Department of Clinical Pharmacy and Toxicology, Maastricht University Medical Center, Maastricht, The Netherlands; 9 Arthritis Center Twente, Medisch Spectrum Twente and University of Twente, Enschede, The Netherlands

**Keywords:** Medication adherence, Inflammatory arthritis, Theoretical Domains Framework, Disease modifying anti rheumatic drugs, Adherence, Non-adherence, Rheumatoid arthritis

## Abstract

**Background:**

Although disease-modifying anti-rheumatic drugs (DMARDs) are the cornerstone of treatment for inflammatory rheumatic diseases, medication adherence to DMARDs is often suboptimal. Effective interventions to improve adherence to DMARDs are lacking, and new targets are needed to improve adherence. The aim of the present study was to explore patients’ barriers and facilitators of optimal DMARD use. These factors might be used as targets for adherence interventions.

**Methods:**

In a mixed method study design, patients (*n* = 120) with inflammatory arthritis (IA) completed a questionnaire based on an existing adapted Theoretical Domains Framework (TDF) to identify facilitators and barriers of DMARD use. A subgroup of these patients (*n* = 21) participated in focus groups to provide insights into their facilitators and barriers. The answers to the questionnaires and responses of the focus groups were thematically coded by three researchers independently and subsequently categorized.

**Results:**

The barriers and facilitators that were reported by IA patients presented large inter-individual variations. The identified barriers and facilitators could be captured in the following domains based on an adapted TDF: (*i*) knowledge, (*ii*) emotions, (*iii*) attention, memory, and decision processes, (*iv*) social influences, (*v*) beliefs about capability, (*vi*) beliefs about consequences, (*vii*) motivation and goals, (*viii*) goal conflict, (*ix*) environmental context and resources, and (*x*) skills.

**Conclusions:**

Patients with IA have a variety of barriers and facilitators with regard to their DMARD use. All of these barriers and facilitators could be categorized into adapted domains of the TDF. Interventions that address individual facilitators and barriers, based on capability, opportunity, and motivation, are needed to develop strategies for medication adherence that are tailored to individual patient needs.

**Electronic supplementary material:**

The online version of this article (doi:10.1186/s12891-016-1289-z) contains supplementary material, which is available to authorized users.

## Background

Inflammatory arthritis (IA) is a long-term autoimmune disease that is characterized by pain, swollen joints, bone damage, and disability [1–3]. The most common conditions of IA are rheumatoid arthritis (RA), ankylosing spondylitis (AS), and psoriatic arthritis (PsA). Inflammatory arthritis, especially RA and PsA, can cause joint damage without proper and early treatment, and early guided treatment with disease-modifying anti-rheumatic drugs (DMARDs) is often recommended [4–8]. The new paradigm in inflammatory arthritis, is to treat to target (T2T), involving regular disease activity monitoring, ideally using the most recently described composite measures and remission criteria [9, 10]. The full benefits of DMARDs can be achieved if patients strictly follow drug regimens [11–14]. However, rates of adherence to prescribed medications in patients with IA are suboptimal and vary widely from 3 to 93 % [15–22]. Therefore, interventions to improve medication adherence are warranted, but current interventions to improve adherence are often complex and not very effective [12, 23]. To discover possible intervention targets, more insights into patients’ motivations to take or not take their medications are essential [24, 25].

**Fig. 1:**
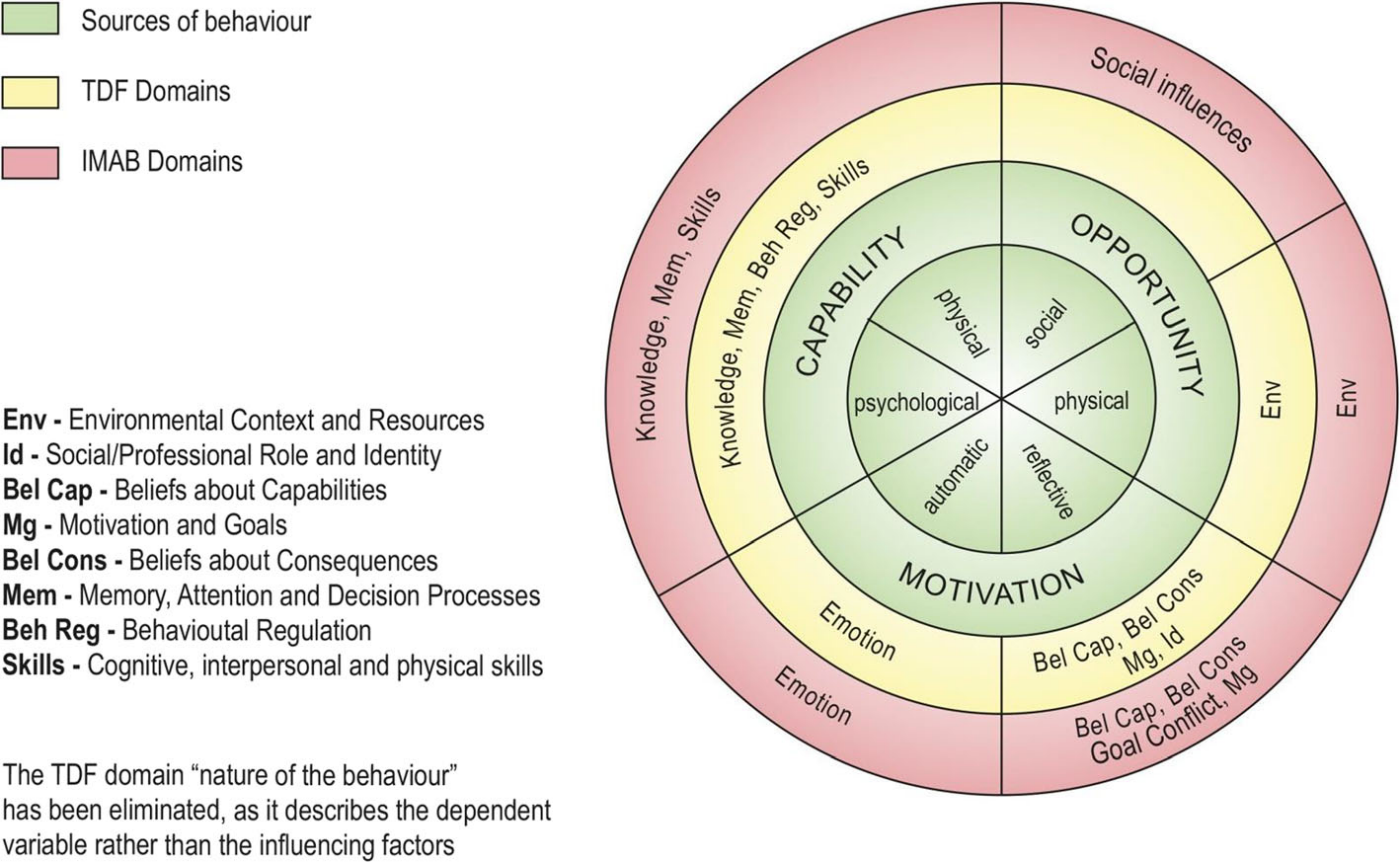
COM-B, derived from the Behaviour Change Wheel [45], TDF [40], and IMAB [25]

Theoretical models may be useful for systematically exploring possible drivers of non-adherence and finding corresponding intervention strategies that target these drivers [26–28]. Although different models have been applied to medication adherence, none of these individual models have been satisfactory for identifying barriers that result in non-adherence [28–33]. A possible explanation might be that the applied models, such as the Health Belief Model [34], Theory of Reasoned Action [35], Theory of Planned Behaviour [36], and Social Cognitive Theory [37], were developed to *understand*, *explain*, or *predict* behaviours. However, to develop useful interventions for adherence, other models or theoretical constructs are needed that are particularly relevant to *changing* behaviour. This means that some essential domains, such as skills [38] or the environment [39], may be overlooked by some theories. To develop a model that contains many domains that could be associated with different behaviour theories, including theories that focus on behavioural change, the Theoretical Domains Framework (TDF) could be helpful. The TDF [40] poses 12 theoretical domains that are based on 33 health behaviour theories (see Additional file 1). The TDF covers a broad spectrum of individual and organisational theories. Thus, it limits the risk of omitting important areas when exploring factors that may impact adherence to DMARD treatment. With regard to medication adherence, the TDF has only been applied in a study of barriers to medication adherence in cardiovascular disease. This resulted in an adapted version of the TDF that contains ten of the 12 original TDF domains [25], the Identification of Medication Adherence Barriers Model (IMAB; see Fig. 1).

To obtain more insights into patients’ drivers that result in adherence and non-adherence, the aim of the present study was to identify both facilitators and barriers of DMARD use in IA patients using an adapted version of the TDF that was designed for medication adherence (IMAB). Although the IMAB was developed to identify medication adherence *barriers*, the present study focused on identifying *facilitators* as well using the same domains of this model.

## Methods

The results of this mixed method study are reported according to the Consolidated criteria for reporting qualitative research (COREQ) [41].

### Design

The study consisted of two phases: (1) a cross sectional survey using a questionnaire that assessed facilitators and barriers of medication use in patients with IA who were using DMARDs, and (2) focus groups with a subgroup of Phase 1 patients to provide in-depth understanding of the identified facilitators and barriers. The aim was to first identify barriers and facilitators of DMARD use in a large group of IA patients by sending out questionnaires and then use these answers in smaller focus group sessions (10–15 patients) to discuss, elaborate upon, and deepen the understanding of the raised barriers and facilitators. until thematic saturation occurred.

### Participants

Patients (*n* = 285) who were registered in the outpatient pharmacy of the Sint Maartenskliniek (Nijmegen, The Netherlands) were invited by mail to participate in this study according to a random sampling method. The inclusion criteria were the following: (1) adult (>18 years old) IA patients who used DMARDs and (2) ability to read and write in Dutch. No exclusion criteria were applied. Both early and established IA patients were eligible to participate in the study. The written invitation consisted of a letter with a description of the study, a questionnaire with open-ended questions, and an invitation to participate in the focus groups. To assess the adherence of the patients to DMARDs, the Compliance Questionnaire on Rheumatology (CQR) was applied. Self-addressed stamped envelopes were provided for the patients to return the questionnaires.

### Procedure

#### Phase 1: questionnaires in patients with IA who used DMARDs

The questionnaire comprised 18 open-ended questions that covered the ten IMAB domains, derived from the TDF (Table 1). The questions were formulated by the researchers (MV, BvdB, and SvD) and pilot-tested through a think-aloud procedure [42] by seven patients with IA, ten people without IA, and two psychologists. The questionnaire allowed the patients to add new domains in cases in which a construct did not fit in any of the existing domains (“Is there anything that influences the use of your medication that is not represented by one of the presented domains?”).

**Table 1:** Summary of questions with corresponding IMAB domains

Domain	Interview question
Knowledge	What would you like to know about your medication to take them (or inject them) as prescribed by your physician?
Emotions	Which emotions are evoked by the use of your medication?
Attention, memory, and decision process	Are you preoccupied with your medication? Do you sometimes, on purpose or accidently, forget your medication? Do you feel sufficiently involved in the decision making process surrounding your medication?
Social influences	In which way does your physician play a role in the use of your medication, other than prescribing them? Does any other health professional (like your pharmacist or your RA nurse practitioner) play a role in the use of your medication? Does your family or do your friends play a role in the use of your medication? Does your work environment (colleagues or supervisors) influence your medication use?
Beliefs about capabilities	In which situations do *you* find it difficult to use your medication?
Beliefs about consequences	According to you, what could be the consequences of taking (or injecting) your medication? According to you, what could be the consequences of NOT taking (or injecting) your medication?
Motivation and goals	What is an important goal (or an important personal motivation) for you to take (or inject) your medication? What is an important goal (or an important personal motivation) for you NOT to take (or inject) your medication?
Goal conflict	What is helpful to you in daily life to be able to take or inject your medication the right way? Which daily hassles prevent you sometimes from taking (or injecting) your medication?
Environmental context and resources	Is the use of your medication sometimes influenced by the ordering, retrieving, delivery, prescribing, and/or reimbursement of your medication?
Skills	According to you, what is important for a patient with RA to be able (physically as well as mentally) to take (or inject) the medication well?

#### Phase 2: focus groups with a subgroup of phase 1 patients

All of the patients who participated in the focus groups had previously completed the questionnaire in Phase 1, and were already sensitized to the research topic and IMAB domains. This so-called sensitization process was meant to enhance the quality and quantity of the patients’ contributions in the later focus group sessions [43]. The patients who accepted this invitation were randomly selected to participate in one of the focus groups. A pilot focus group was not conducted, since patients were involved in the development of the questions and sensitized by filling out the questionnaire. The duration of the focus group sessions was 2 h, and they were conducted at Sint Maartenskliniek. Two researchers led each focus group. In the first focus group, one female researcher (AH) facilitated plenary conversations, and another female researcher (MV) studied the group process and took notes. Two breakout sessions were led by two female researchers (MO and AH). The second focus group was organised similarly, with the exception that the breakout sessions were led by one female researcher (AH) and one male researcher (BvdB). After introducing all of the participants in the focus group, the aim of the group discussion was clarified, namely to identify potential barriers and facilitators of DMARD use. After the domains of the model were introduced and explained, the group was divided into two breakout groups. Each subgroup was given 45 min to explore five of the ten domains of the IMAB, with a focus on identifying barriers and facilitators for each domain. In the plenary session, after the breakout session, all of the barriers and facilitators that were identified in the subgroups were discussed. The patients were then asked if they could think of any additional barriers or facilitators that did not fit in one of the presented domains, such that new domains could be added.

### Analysis

Both focus groups were audiotaped with consent from all of the patients and transcribed verbatim. Descriptive statistics were computed (means, standard deviations, and frequencies where appropriate). To ensure the patients’ privacy, all identifying information was anonymised. All patients in the focus group read the transcripts and were given the opportunity to discuss changes they felt were needed before coding the transcripts (member check). The answers of the questionnaire and the content of the transcripts was then thematically analysed [44] to identify themes and subthemes that reflected facilitators and barriers for adherence and non-adherence. The notes that were taken during the breakout sessions were also analysed. Three researchers (MV, JV, and BvdB) independently coded the data and categorized the codes into themes and subthemes using the IMAB model. The coding process was discussed until consensus was reached among the researchers.

To facilitate a future implementation process, the barriers and facilitators that were identified with the corresponding IMAB domains were categorized according to the components of the Behaviour Change Wheel [45]: Capability, Opportunity, and Motivation (COM). In this behaviour system, these three components interact to generate Behaviour (COM-B; Fig. 1).

## Results

One hundred twenty of the 285 patients (42 %) returned the questionnaire The mean age of the patients that returned the questionnaire was 59.6 years (SD = 15.4 years), and 60 % of the patients were female. Most of the patients had RA (71 %). DMARD adherence according to the CQR was 68 % (Table 2).

**Table 2:** Patient characteristics

	Patients (*n* = 285)^a^
Age, years (SD)	59.6 (±15.4)
Female, *n* (%)	171 (60 %)
Diagnosis:	
Rheumatoid arthritis	201 (71 %)
Rheumatoid factor (RF positive+)	140 (70 %)
Anti-citrullinated Protein antibodies Positive (Anti-CCP+)	114 (54 %)
Psoriatic arthritis	49 (17 %)
Juvenile idiopathic arthritis	12 (4 %)
Other diagnoses	23 (8 %)
Pharmacological therapy, *n* (%)	
Methotrexate	239 (84 %)
Sulphasalazine	7 (2 %)
Leflunomide	8 (3 %)
Hydroxychloquine	52 (18 %)
Glucocorticosteroid	29 (10 %)
Biological DMARDs	81 (28 %)

^a^Questionnaires were processed anonymously. The patient characteristics that are presented in the table were gathered from the patients that filled out the questionnaire

The subgroup of patients in the focus groups was comparable with regard to mean age (59.44 years, SD = 9,4 years). Of the patients in the focus groups, 85 % were female, and most of the patients had RA (88 %). DMARD adherence according to the CQR was 43 %.

Table 3 summarizes the identified barriers and facilitators for IA patients (by the respondents of the questionnaire and the participants of both focus groups) with regard to DMARD use, categorized according to the IMAB model and COM-B core components that were derived from the Behaviour Change Wheel [45]. The Attention, Memory, and Decision Process domain was split into two separate domains based on consensus between the three researchers (“Memory and Attention” and “Decision-making process”) to accommodate the categorization of all of the identified barriers and facilitators. Several of the identified barriers and facilitators were categorized into more than one IMAB domain because of the multifaceted nature of these factors. All of the barriers and facilitators that were collected could be categorized in the presented domains. No new, additional domains were mentioned by the focus groups, which supported the data received from the respondents of the questionnaire.

**Table 3:** IMAB domains and corresponding facilitators and barriers categorized according to COM-B components

COM-B	Domain	Facilitators	Barriers
Capability	Knowledge	Knowledge of treatment effect Information about necessity Information about alternative medication Information about experiences from others Knowledge of how to administer medication	Knowledge of side effects Knowledge of cost of medication
	Skills	Cognitive abilities Coping strategies Communication skills Fine motor skills	Insufficient cognitive, communicative, or physical skills to understand and/or administer medication
	Memory and Attention	Treatment effect Aids (to remember) Social support Embedded in daily routine	Lack of daily routine Experiencing side effects Forgetting to take medication Change of appearance of medication Impact on lifestyle Lack of treatment effect
	Decision-making process	Self-management (patient decides) Satisfying relationship with health professional (communication and trust)	Lack of involvement of health professional (health professional decides, no shared decision making) Doubting own knowledge Influence of health insurance companies
Opportunity	Environmental context and resources	Logistics (having medication in stock) Access to health professionals	Quality of product (needle) Logistics (medication storage temperature, pharmacy has no stock) Cost of medication Travelling (clearing injectable medications through customs) Change of name or appearance of medication
	Social influences	Health professionals (rheumatologist, pharmacist, nurse, general practitioner): capabilities, trust, and empathy Health insurance company: reimburses medication, provides clear information Family and friends: support adherence, support in choices of medication, instrumental support Colleagues: support, understanding	Lack of support from colleagues (incomprehension, negative reactions)
Motivation	Beliefs about capabilities	Aids (to use, to remember) Self-efficacy Good (overall) health status	Difficulty in adherence because of social and work events Lack of daily routine Worse health status Difficult to inject Experience of side effects Doubting own knowledge with regard to medication (as barrier to adherence)
	Beliefs about consequences	Belief of treatment effect Belief of being able to better participate (social, work)	Experience of (long- and short-term) side effects Belief that medication will be harmful: higher disease activity Lack of belief in efficacy Dependency on medication Less acceptance of (long-term) medication Non-acceptance of diagnosis
	Emotions	Joy Gratitude Hope Confidence	Anxiety Sadness Anger Dependency on medication Irritation Incomprehension Disparity Powerlessness/helplessness Insecurity Feeling overwhelmed Feeling crestfallen Grief Embarrassment Regret Stress Disappointment Desolation Despair Agitation
	Motivation and Goals	Improvement of Quality of Life Treatment effect Social participation (including work) Improvement of life expectancy Better relationship with health professional Maintain autonomy	Worse health status/wellbeing Side effects Comorbidity Complexity of regimen Resistance of need for medication Denial of existence of illness Difficulty administering medication Lack of daily routine
	Goal conflict	Embedded in daily routine Treatment effect leading to active social participation (e.g., work) Instrumental support (aids, information) Social support Method of administration (intravenous or low frequency) Stock (always available)	Experiencing side effects Restrictions due to using medication (no alcohol) Worse well-being (mentally and/or physically) Not able to participate (social, work) Distracted from taking medication Lack of social support

### Capability

Capability, the first COM-B component, reflects the individual’s psychological and physical capacity to engage in the activity concerned [45]. Capability encompassed the following domains: Knowledge, Skills, Memory and Attention, and Decision-making Process.

#### Knowledge

Knowledge about DMARD use was considered both a barrier and a facilitator of adherence. Information about how to administer the medication, general information (also about alternative medications), and information about necessity and the time to onset of effect of DMARD use were mentioned frequently as facilitators of DMARD use. Knowledge of side effects and the cost of some of the medications were perceived as barriers. Some patients sometimes felt guilty using expensive medications, and they felt that they were a burden to society. Thoughts and doubts about how precisely the medication acts on the body were also mentioned as a potential barrier:

*“….You do not know how these medications exactly act in your body. Yes, they tend to decrease the disease activity, but you need to know how the medication works. It maybe goes beyond my knowledge, but surely it must be achievable to translate this information in a simple way. I think that would help to accept that you can take or inject this medication…”.* Female, 63 years, diagnosed with RA in 2015

Clear and understandable instruction inserts were found to facilitate adherence.

#### Skills

Patients mentioned that having acquired appropriate coping skills to manage the disease and its medication were facilitators for medication adherence. Examples were given, such as being able to accept the fact that they had to use their medication probably for the rest of their lives. Cognitive abilities were also reported by the patients as facilitators, such as being able to dose their medication, understand which day of the week they need to take certain medications, or being able to distinguish between different medications. Lacking these abilities was seen as a barrier to medication adherence. Several barriers, such as how to open the package of the medication or difficulties with opening the tap to get water for medication, were also mentioned. These physical abilities were referred to as “fine motor skills.” Communication skills were considered to be crucial, such as being able to communicate in a timely manner with the rheumatologist about side effects when these side effects prevented patients from using the prescribed medication.

#### Memory and attention

Patients mentioned that incorporating the intake of their medication in their daily routines was a great help. Aids, such as an alarm on their smartphone or help from a family member to remind them to take the medication, were sometimes used. Experiencing beneficial effects of the medication facilitated adherence to medication use.

*“They prescribed it, I took it and it went very well. Then I have something to hold on to. If I continue, then all will be great. That is what happened.”* Female, 75 years, diagnosed with RA in 2009

Social support was mentioned as a facilitator as well. Support from family, friends, and colleagues was considered very essential by all of the patients.

Several barriers of DMARD use were mentioned by the patients, including the absence of a daily routine, experiencing side effects of the medication, forgetting to take the medication, and a lack of efficacy of the medication. Another barrier was related to the appearance or name of the medication. For example, changing the colour or name of the medication was considered confusing and potentially dangerous. The patients also considered the impact of the medication as intruding on their lifestyle, such as not being able to consume alcohol on certain days because of the medication, which was felt as patronizing and limiting social activities.

#### Decision-making process

The patients mentioned that the physician plays an important role as a facilitator for adherence, specifically in the decision-making process. A good relationship with the treating physician was considered crucial for trust and communication about disease management, including adherence. The patients reported that if they have self-management skills, then a shared decision-making process was considered a natural process, subsequently leading to a more equal relationship with the physician and better adherence. However, potential barriers to adherence were mentioned by the patients, such as a lack of interest and lack of competence by the physician to involve the patient in the treatment regimen or a lack of competence and lack of interest by the patient, which would hamper the patient’s involvement as a partner in the treatment.

*“You need to be taken seriously. A little bit of interaction. Of course you need to trust the abilities of your physician, but it wouldn’t hurt to be critical in a positive way…”* Male, 47 years, diagnosed with RA in 2011

The influence of health insurance companies was also mentioned as a potential barrier by the patients, specifically the possibility of a changing reimbursement policy that may influence the adherence or non-adherence to DMARDs.

### Opportunity

Opportunity, the second COM-B component, reflects all of the factors that lie outside the individual that make the behaviour possible or prompt it [45]. Opportunity encompassed the following domains: Environmental Context and Resources and Social Influences.

#### Environmental context and resources

Access to the rheumatologist when needed was felt as an essential facilitator for the patients. Another facilitator that was discussed by the patients was logistics. The prescribed medication should be in stock in the pharmacy. Logistics was also mentioned as a barrier in cases in which the pharmacy did not have the medication in stock. Another barrier was the quality of the products, such as the needles for injections. The patients complained that the needles were too blunt. Additionally, clearing injectable medications through customs while travelling abroad and situations in which the storage temperature could not be controlled were also perceived as barriers.

*“Going on holiday, taking your injections with you, and the temperature should not exceed above 25 C, that is a disaster. Then I think: the pharmacist should provide a little cool box with these kind of medications”*……Female, 63 years, diagnosed with RA in 2015

The cost of medication was also mentioned as a barrier, but the patients stated that they would be willing to pay a small amount for the medication if it was not covered by their insurance in order to obtain them.

#### Social influences

Support from the social environment of the patient was mentioned as a facilitator. One example that was related to the private environment was mentioned in one of the focus groups:

*“Sometimes, when I am feeling good, I think, ‘I just skip my medication.’ but my husband always pushes me: ‘You have to take your medication just as usual.’ But, still, I find it difficult (to take my medication always)…”* Female, 64 years, diagnosed with RA in 2000

Therefore, the patients reported that support from family and friends could be helpful to stimulate adherence. This support could be provided by discussing treatment options or by giving instrumental support, such as helping administer the medication.

The patients experienced an understanding of their disease from their social network. They indicated that they received support from their work environment, especially when they showed an open and transparent attitude with regard to their condition. Concerning their treating health professionals (i.e., physician, pharmacist, nurse, and general practitioner), their capabilities, mutual trust, and empathy were considered crucial for treatment. Especially in long-term conditions, bonding with health professionals was found by the patients to be beneficial and recommended with regard to adherence. Furthermore, reimbursement and clear information about the medication that is provided by the health insurance companies were mentioned as facilitators of adherence. As a barrier, the patients mentioned a lack of support, specifically incomprehension by colleagues and subsequent negative reactions.

### Motivation

Motivation, the third COM-B component, reflects brain processes that energize and direct behaviour [45]. Motivation encompassed the following domains: Beliefs about Capabilities, Beliefs about Consequences, Emotions, and Motivation and Goals.

#### Beliefs about capabilities

This domain represents the patients’ *beliefs* about their own capabilities, such as believing they are able to manage their condition (self-efficacy). Having good overall health status (apart from IA) and using aids (to remember to take their medication) were mentioned as facilitators to strengthen their belief in their ability to use DMARDs. Social and work events, a lack of a daily routine, worse health status, difficulties administering the medications (such as how to inject them), experiencing side effects, and doubting their own knowledge about their medications were mentioned as barriers.

#### Beliefs about consequences

Strong beliefs about the beneficial effects of treatment and believing that they are able to participate better in social activities and work were mentioned as facilitators to medication adherence. The following items were mentioned as barriers: fear, feelings of doubt of efficacy of medication, feelings of desire to stop treatment to be free of the ongoing dependency on medication, feelings of insecurity about the long-term effects, and non-acceptance of the IA diagnosis.

#### Emotions

In contrast to the basic negative emotions (fear, anger, and sadness) and other negative feelings (Table 3) that were often mentioned as barriers to medication adherence, positive emotions (joy, feelings of gratitude, hope, and confidence) were rarely mentioned with regard to DMARD use.

*“I am worried about my organs, such as my heart, my liver and my kidneys. It is toxic, everything I need to take. I cannot imagine that my body can tolerate it for many years. So, that is my biggest fear….”* Female, 51 years, diagnosed with RA in 2014

#### Motivation and goals

Facilitators of adherence that were mentioned in this domain, were experiencing a positive effect of the medication and being able to maintain autonomy, social participation, and work participation, leading to improvements in quality of life. The patients were well aware that treatment options have significantly better health outcomes these days, which motivates them to take their medications. Moreover, being able to hold grandchildren, being able to do volunteer work, or being able to keep their jobs were essential facilitators as well. Patients were taking their medication to please their physician to strengthen the relationship. A longer life expectancy was also considered a facilitator with regard to adherence. Patients expressed feeling resistant toward taking their medication, especially patients who already felt down because of the long-term nature of their condition. More practical issues surfaced as barriers, such as a lack of a daily routine, worse health status (not feeling well, besides the IA), fear of side effects, and difficulty administering the medication (use of injections). Especially the elderly patients, expressed that their regimen was sometimes perceived as too complex, resulting in non-adherence.

Goal conflicts arose in situations in which the taking of DMARDs interfered with the patients’ valued goals. The barriers that were identified with regard to goal conflict included experiencing side effects (and therefore sometimes not being able to participate socially or in a work environment), not being able to consume alcohol while taking their medication (and therefore sometimes feeling restricted in social contexts), not feeling well physically and mentally (besides the IA) and therefore not willing to take the medication, perceiving a lack of social support, and being distracted from taking the medication. Especially during holidays, of which the goal is to relax and enjoy, when there is a lack of a routine, adhering to treatment was found to be difficult, and could lead to ambivalent feelings:

*“…I always eat my breakfast before the intake of my medication. During holidays, the routine is gone, so a holiday is always positive and nice, but also a little bit negative….”* Male, 51 years, diagnosed with Psoriasis and RA in 2001

However, some facilitators accommodated the intake of DMARDs without interfering with the patients’ goals. When taking the medication was firmly embedded in a daily routine, it was easier to adhere to DMARD use. Instrumental support, such as aids (week boxes or a phone alarm), to stimulate adherence was considered helpful. Social support from family, friends, health professionals, and colleagues also strengthened adherence behaviour. Social relatives may emphasize the importance of continuing to use the medications despite interfering with a goal that is set by the patient and especially the long-term benefits. Patients mentioned that an easy way to administer their medication, such as intravenously, or a low frequency of use, helped them to be adherent without causing goal conflicts. The less effort the patient has to expend is associated with a higher probability of adherent behaviour.

## Discussion

The barriers and facilitators that were reported by IA patients in the present study presented large inter-individual variations. The barriers and facilitators were identified using the following domains of the existing adapted version of the TDF for medication adherence [25, 40]: (*i*) knowledge, (*ii*) emotions, (*iii*) attention, memory, and decision processes, (*iv*) social influences, (*v*) beliefs about capability, (*vi*) beliefs about consequences, (*vii*) motivation and goals, (*viii*) goal conflict, (*ix*) environmental context and resources, and (*x*) skills. These domains, originally developed to identify medication adherence barriers, appear to be useful for also identifying medication adherence facilitators. In the present study, in addition to using the adapted domains of the TDF, the Behaviour Change Wheel helped categorize all the identified barriers and facilitators under the three elements of the Behaviour Change Wheel (COM-B). Patients should have the *opportunity* to take their medication, should be *motivated* to take their medication, and be *capable* of taking their medication. Because the Behaviour Change Wheel can be effectively used in a process of designing and implementing intervention tools [45], categorization of the identified barriers and facilitators using the domains of an adapted TDF and the Behaviour Change Wheel elements may enhance the future development and implementation of an intervention for adherence.

Most facilitators and barriers that were found in the present study were comparable to factors that were related to adherent and non-adherent behaviour in other studies of long-term diseases. Two recently published reviews summarized quantitative data on facilitators and barriers in patients with RA [16, 26], and some similarities with the present results were found. A good patient-health professional relationship, knowledge about treatment, the absence of negative effects, an easy treatment regimen, patients’ belief that they are able to fulfil the therapy, and an appropriate amount of information that is provided by the healthcare provider appear to improve adherence. Consistent with these two previous reviews, barriers that resulted in non-adherence in the present study included not believing in the necessity of antirheumatic medication, a busy lifestyle, and receiving contradictory information from the healthcare provider. In contrast, the finding that poorly developed health services with inadequate or non-existent reimbursement may stimulate non-adherent behaviour was not evident in our study. The latter might be explained by the fact that good health insurance is available to all patients in The Netherlands.

According to our qualitative study, barriers and facilitators that were mentioned by the patients as related to adherence and non-adherence but were found not to be statistically significant in the previous reviews were coping, immediacy of side-effects, perceived effectiveness of the medication (outcome expectation), lack of belief in benefit, number of medications, level of Methotrexate dose, change in appearance of the medication, social support, and improvement in quality of life.

One explanation for the different findings between the present study and the previous reviews may be related to the type of study (i.e., qualitative vs. quantitative research). In the present qualitative study, the patients were free to write down their own experiences as answers to open-ended questions. In the focus groups, discussions that were based on individual opinions were held freely, which contributed to a variety of answers and reflected barriers and facilitators to medication adherence. The previous reviews consisted of quantitative studies, in which different factors were mentioned as statistically related or unrelated to adherence and non-adherence.

The present study provided an overview of many different barriers and facilitators with regard to DMARD use in IA patients. Such various opinions were attributable to the fact that we used an adapted Theoretical Domain Framework with all its domains, thus offering patients the opportunity to identify many different factors that may influence their adherence and non-adherence. Most of the patients’ barriers and facilitators were captured by the TDF, which may contribute to developing useful, effective, tailor-made interventions for individual IA patients to stimulate adherence.

All barriers or facilitators that the patients mentioned could be categorized under one of the domains of the adapted TDF, suggesting that this framework is helpful for identifying barriers and facilitators of medication adherence. Another strength of the TDF is the combination of cognitive and behavioural constructs with appropriate implementation strategies from the Behaviour Change Wheel [45]. The Behaviour Change Wheel comprises three elements—capability, opportunity, and motivation—that may help identify types of interventions that may be suitable for addressing the barriers and facilitators of medication adherence in future studies.

A few limitations of the present study should be mentioned. First, selection bias could have occurred because non-adherent patients might have been less willing to participate in the study. To determine whether selection bias occurred, all of the respondents (*n* = 120) completed the CQR to assess medication adherence. The rate of adherence was 67.5 %. This represents a comparable rate of adherent and non-adherent respondents compared with previous studies on medication adherence in RA, which limits the possibility of selection bias [15–17].

Second, the response rate for the questionnaire was modest, 42 %. A possible explanation for this modest response could be that it was rather time consuming to complete the questionnaire with open-ended questions and the CQR. Because of the applied methodology, it was not possible to examine whether responders of the questionnaire differed from the non-responders in this study nor whether this could have influenced our findings. This approach was chosen to guarantee anonymity, to emphasize the aim of this study to identify barriers and facilitators to DMARD use, and to keep the already long questionnaire feasible to fill out.

Third, the present study may have limited generalizability. We only included Caucasian patients from one region in The Netherlands. None of the participants were from other ethnic backgrounds or represented various religions. Therefore, potential difficulties in taking medications because of cultural or religious reasons (e.g., Ramadan) were not mentioned in this study. Future studies should include patients with different ethnicities and different religions and from different regions to confirm the present findings.

Fourth, the domains that were used in the present study were previously established. One point of criticism may be that the domains that were introduced to the patients may have led them to specific responses. However, in the design of our study, we anticipated this possible shortcoming and added an extra question: “Is there anything that influences the use of your medication that is not represented by one of the presented domains?” Some of the patients mentioned additional barriers and facilitators, but these could be categorized under one of the existing domains. The fact that all of the barriers and facilitators could be categorized into one of the domains of the TDF indicates the usefulness of this framework for capturing all barriers and facilitators that are important for IA patients in the use of DMARDs.

Fifth, patterns or relationships between the theoretical domains of the TDF are not specified within the TDF framework, in contrast to other theoretical models of behaviour, such as the Theory of Planned Behaviour [36]. The use of the TDF framework in the present study was specifically meant to capture as many barriers and facilitators as possible with regard to adherence, without exploring the relationships between domains or weight of individual domains. Future studies should explore these relationships and their relative weight with regard to barriers and facilitators of medication adherence. Because we used a different approach, the present study was not an attempt to replace existing behavioural theories.

## Conclusions

The patients in the present study identified a large variety of barriers and facilitators of DMARD use. All the identified facilitators and barriers fit in the domains of an existing adapted version of the TDF. Therefore, the TDF appears to be an appropriate framework for systematically assessing drivers that influence adherence and non-adherence to the use of DMARDs. However, further research is necessary to provide insights into (*i*) the frequency and impact of different barriers and facilitators on adherence, (*ii*) the development of tools to detect facilitators and barriers in individual patients, and (*iii*) the extent of modifiability of barriers and reinforcement of facilitators. This is essential before an intervention can be designed, implemented, and evaluated for IA patients with regard to DMARD use.

### Practice implications

The present study was one step toward developing an intervention to optimize DMARD use in IA patients. Given the fact that the nature of the identified facilitators and barriers of medication adherence is very heterogeneous, interventions that improve adherence should consist of an inventory of patient’s individual barriers and facilitators, followed by an intervention that is tailored to individual patient needs. The Behaviour Change Wheel may be beneficial as a guiding model in this process. Furthermore, the present study shows that the TDF framework is a useful tool for assessing patients’ individual facilitators and barriers.

## Additional file

Additional file 1
**Appendix 1.** Theories used in the Theoretical Domains Framework (DOCX 15 kb)

## Abbreviations

AS: Ankylosing spondylitis
CQR: Compliance Questionnaire on Rheumatology
DMARDs: Disease-modifying anti-rheumatic drugs
IMAB: Identification of Medication Adherence Barriers Model
PsA: Psoriatic arthritis
RA: Rheumatoid arthritis
TDF: Theoretical Domains Framework
TDF: Theoretical Domains Framework
