# Supplementary material for: Barriers and facilitators to disease-modifying antirheumatic drug use in patients with inflammatory rheumatic diseases: a qualitative theory-based study
Source: BMC Musculoskelet Disord. 2016 Oct 21;17:442. doi: 10.1186/s12891-016-1289-z (PMC5075197; doi:10.1186/s12891-016-1289-z)
Supplement: Additional file 1 — Appendix 1. Theories used in the Theoretical Domains Framework (DOCX 15 kb) [file 12891_2016_1289_MOESM1_ESM.docx]

**Supplementary material**

Appendix 1. Theories used in the Theoretical Domains Framework.

MOTIVATION THEORIES

• Theory of planned behaviour (+ theory of reasoned action)

• Protection motivation theory, health belief model

• Social cognitive theory

• Locus of control theories

• Social learning theory

• Social comparison theory

• Cognitive adaptation theory

• Social identity theory

• Elaboration likelihood model

• Goal theories

• Intrinsic motivation theories

• Self‐determination theory

• Attribution theory

• Decision-making theories (e.g., social judgment theory, “fast and frugal” model,

systematic *vs*. heuristic decision making)

• Fear arousal theory

ACTION THEORIES

• Learning theory

• Operant theory

• Modelling

• Self‐regulation theory

• Implementation theory/automotive model

• Goal theory

• Volitional control theory

• Social cognitive theory

• Cognitive behaviour therapy

• Transtheoretical model

• Social identity theory

ORGANISATION THEORIES

• Effort‐reward imbalance

• Demand‐control model

• Diffusion theory

• Group theory (e.g., group minority theory)

• Decision-making theory

• Goal theory

• Social influence

• Person situation contingency models
